# Endoscopic Powered Intracapsular Tonsillectomy and Adenoidectomy in Pediatric Obstructive Sleep Apnea With High-Risk Comorbid Disease Conditions: A Case Series

**DOI:** 10.7759/cureus.61621

**Published:** 2024-06-03

**Authors:** Masao Noda, Ryota Koshu, Mari Dias, Chizu Saito, Makoto Ito

**Affiliations:** 1 Otolaryngology - Head and Neck Surgery, Jichi Medical University, Shimotsuke, JPN

**Keywords:** high-risk condition, surgery, sleep, adenoidectomy, intracapsular tonsillectomy, endoscopy, obstructive sleep apnea, pediatrics

## Abstract

Objective: Pediatric obstructive sleep apnea (OSA) caused by adenoids or an enlarged palatine tonsil has a negative impact on physical and mental growth. Surgical removal of the tissue is effective but entails a life-threatening risk of postoperative bleeding, which is up to 30 times higher in chronic pediatric disease cases. However, endoscopes and resection devices provide safe, reliable surgical methods. Here, we report the efficacy and safety of endoscopic powered intracapsular tonsillectomy and adenoidectomy (PITA) for pediatric OSA in patients with high-risk comorbidities.

Methods: This retrospective case series included pediatric patients with OSA who underwent PITA at a single tertiary medical center between April 2017 and May 2023. Ten patients (three males and seven females; mean age 6.4 years, range 2-12 years) were included; all met the Japanese criteria for complex chronic pediatric conditions.

Results: The average operative time was 61 min; a microdebrider was used in eight cases and a coblator in two cases. Although there was no postoperative bleeding, one case experienced regrowth.

Conclusions: Our data show that an endoscopic PITA approach could reduce the risk of severe bleeding and relieve the sleeping conditions of pediatric patients with complex chronic OSA.

## Introduction

Pediatric obstructive sleep apnea (OSA) is often caused by adenoids and hypertrophy of tonsils and has a significant impact on physical and mental growth [1,2]. Compared with adults, children experience more significant airway obstruction from adenoids and palatine tonsils due to facial size and face a variety of burdens depending on the severity of the condition. Surgical removal of the tissue is an effective treatment, based on a comprehensive consideration of the severity of the apnea, size of the tonsils and adenoids, age and body size, and complications. However, postoperative pain and bleeding risks should be considered, especially in children [3,4]. Cases of death due to airway obstruction or blood loss due to hemorrhage have been reported, especially in children with chronic diseases, where the risk of death is approximately 30 times higher than that in children without any disease. Surgery may not be performed or may be postponed in children with chronic diseases due to the aforementioned postoperative risks of death as part of perioperative management [5,6].

In recent years, technological developments have led to the development of endoscopes and resection devices that provide safer and more reliable surgical methods [7,8]. Endoscopic powered intracapsular tonsillectomy and adenoidectomy (PITA) is also available for tonsil surgery, and our institution has reported its safety and efficacy compared to those of conventional tonsillectomy [9]. There have been no reports to date on tonsil surgery focusing on chronic pediatric diseases.

In this case series, we present the high-risk cases of endoscopic PITA in pediatric patients with complex chronic conditions (CCCs).

## Materials and methods

Patients

This was a retrospective case series of pediatric patients with OSA with high-risk comorbidities who underwent PITA at a single tertiary medical center (Jichi Medical University Hospital, Shimotsuke) between April 2017 and January 2023. All patients were organized by age, gender, comorbid disease, apnea-hypopnea index (AHI) or respiratory event index (REI), duration of hospital stay, Grade of tonsil hypertrophy, devices used, operative time, presence of postoperative bleeding, and presence of regrowth. Written informed consent was obtained from the participants’ parents.

The pediatric CCC classification system includes diseases in the ICD-10 domain, such as neurological, cardiovascular, and genetic abnormalities, with an expected duration of at least 12 months (unless death occurs) and involving either several separate organ systems or one organ system, severe enough to require specialty pediatric care and probably some period of hospitalization in a tertiary care center. Patients who met the criteria for pediatric chronic diseases in Japan were selected by two specialists with more than 10 years of experience. The case data were reviewed retrospectively, and comorbidities and perioperative and postoperative outcomes were summarized.

Surgical method

The surgery was performed as described in the literature [9], using either a microdebrider or coblator as the device, and considering the patient’s age and bleeding risk. The microdebrider was a radar blade attached to a 40-degree curved tip, used at a setting of 600-800 rpm. A coblator (Coblator 2 surgery system, Smith+Nephew, England) with PROcise™ MAX was used to reduce intraoperative bleeding, using coblate mode with output at 5-7. Over 90% of the palatine and adenoid tissues were removed, and hemostasis with bipolar diathermy was performed at the end of surgery. Regarding the choice of device, the surgeon makes a comprehensive decision, using the coblator in cases where the patient is younger than two years old, in cases at high risk of bleeding, or in cases where postoperative management is considered difficult due to bleeding.

## Results

Ten children with chronic disease comorbidities are included in Table 1.

**Table 1:** Patient background and outcomes of 10 cases with chronic disease comorbidities who underwent PITA surgery LV-RA: Left ventricular-right atrium; PDA: patent ductus arteriosus; PFO: patent foramen ovale; PH: pulmonary hypertension; VSD: ventricular septal defect; PITA: powered intracapsular tonsillectomy and adenoidectomy

Patient No.	1	2	3	4	5	6	7	8	9	10
Age(years)	9	11	4	2	3	4	4	6	8	12
Sex	Female	Female	Female	Male	Female	Male	Female	Female	Female	Male
Comorbid disease	Trisomy21, asthma, VSD	Mitochondrial respiratory chain disorders, cerebral palsy, SCN2A-related disorder	Turner syndrome, hypothyroidism, Coarctation of the aorta	Ebstein anomaly, congenital esophageal atresia, developmental retardation	Crouzon syndrome, Chiari malformation	biliary atresia splenic malformation syndrome	lissencephaly, Miller Dieker syndrome	Trisomy21, asthma, VSD, pseud LV-RA communication PFO, PH	Trisomy21, asthma, VSD, PDA	cerebral palsy, Neonatal asphyxia
Duration in hospital (day)	8	9	12	7	8	10	9	8	9	8
AHI (/hour)	18	63	132.7	-	16	-	52	16	12.1	-
Grade of tonsil hypertrophy	3	3	4	2	3	3	4	4	3	2
Device	Microdebrider	Microdebrider	Microdebrider	Coblator	Microdebrider	Microdebrider	Coblator	Microdebrider	Microdebrider	Microdebrider
Postoperative hemorrhage	-	-	-	-	-	-	-	-	-	-
Operative time (min)	52	51	73	33	70	74	57	69	36	68
Regrowth	-	-	-	-	-	-	-	-	+	-

The patients comprised three boys and seven girls with a mean age of 6.4 (2-12) years. A microdebrider was used in eight cases and a coblator in two cases. The average operative time was 61 min; there was no postoperative bleeding in any of the cases, although re-enlargement occurred in one case. Two representative cases are shown below.

Case: patient 2

An 11-year-old girl was bedridden due to underlying medical conditions after visiting our pediatric department for an SCN2A abnormality, cerebral palsy, and intractable spasticity. Since infancy, the patient had upper airway stenosis and was treated with bilevel positive airway pressure (BiPAP); however, three months had passed with worsening of the upper airway stenosis sounds. Considering the difficulty of continuous BiPAP management, the patient was referred for surgical treatment.

Physical Examination and Findings

**Figure 1:**
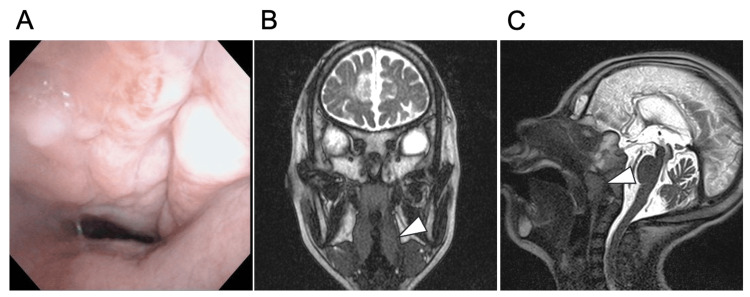
Physical examination and findings of patient 2 A: Nasopharyngeal fiber scopy showed pharyngeal stenosis, particularly at the level of the oropharynx. B and C: MRI (B: coronal section, C: sagittal section) shows tonsil and adenoid hypertrophy (white triangle).

The patient had grade 3 palatine tonsils (Brodsky), a small forehead, and an overhanging tongue root, making it difficult to observe the inferior pole of the tonsils [10]. Nasopharyngeal fiberoptic examination showed pharyngeal stenosis, particularly at the level of the oropharynx (Figure 1A). The patient also had tonsillar and adenoid hypertrophy (Figures 1B, 1C), and simple polysomnography (PSG) showed a REI of 63.1, leading to the decision to provide surgical treatment.

Surgery and Postoperative Course

**Figure 2:**
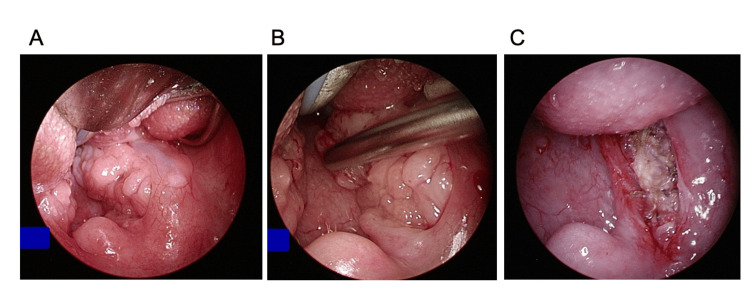
Surgery procedure of patient 2 PITA was performed with a microdebrider, a Radenoid blade attached to a 40-degree curved tip. While using a 0-degree rigid endoscope, resection was performed to leave a return membrane of the palatine tonsils and remove at least 90% of the tissue. Before (A), during (B), and after (C) the resection. PITA: Powered intracapsular tonsillectomy and adenoidectomy

The surgical procedure has been described previously [9]. PITA was performed with a microdebrider and a RADenoid™ blade attached to a 40-degree curved tip at a setting of 600-800 rpm. Using a 0-degree rigid endoscope, resection was performed to leave the tonsillar capsule of the palatine tonsils and to remove at least 90% of the tissue (Figure 2).

The adenoids were resected using a microdebrider under a 70-degree rigid endoscope. Hemostasis of the tonsils and adenoids was performed using bipolar diathermy at the end of the surgery.

The patient had an uneventful postoperative course and was discharged seven days after surgery. BiPAP was not required one year after surgery. Thereafter, the patient wore the BiPAP device only at night from one year onwards.

Case: patient 5

A three-year-old girl had been snoring since birth and was diagnosed with OSA at the otolaryngology department of another hospital. Surgery was not performed because of perioperative risks and other complications. Two years and six months later, the patient developed worsening snoring symptoms and visited the pediatric department at our hospital. During the course of the disease, her breathing worsened during the night, and she could not be fitted with a nasal airway or BiPAP device; therefore, she was referred to our pediatric otolaryngology department.

Her medical history included Crouzon syndrome and Chiari malformation, for which cranioplasty was performed in the plastic surgery department of the hospital.

Physical Examination and Findings

**Figure 3:**
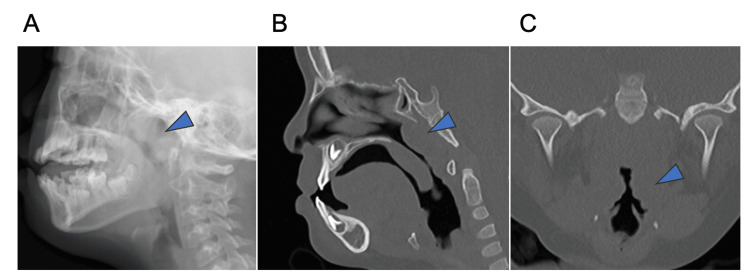
Physical examination and findings of patient 5 X-ray (A) and CT-scan (B: sagittal section, C: coronal section) show adenoid and tonsil hypertrophy (blue triangle).

**Figure 4:**
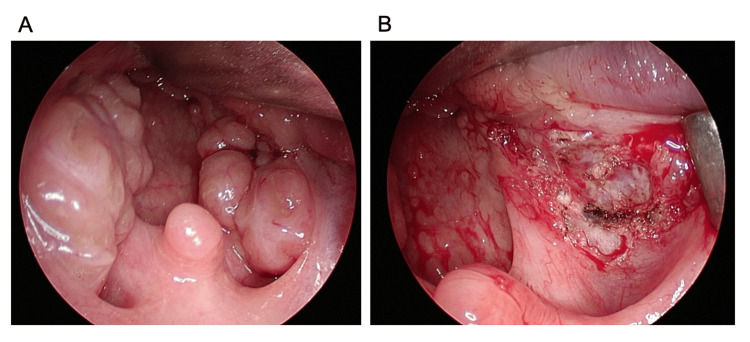
Surgical image of patient 5 PITA was performed with a microdebrider for patient 5. The endoscopic image shows before (A) and after (B) the PITA surgery. PITA: Powered intracapsular tonsillectomy and adenoidectomy

The patient had grade 3 palatine tonsils (Brodsky), and imaging findings showed adenoid hypertrophy. Facial deformity and a small aperture made it difficult to visualize the lower pole of the tonsil from the outside (Figure 3). PSG showed an AHI of 16.0. PITA was performed using a microdebrider as described in this case (Figure 4).

The patient had an uneventful postoperative course and was discharged seven days after surgery. Postoperatively, her snoring symptoms improved, and she had less anxiety about perioperative respiratory management during other surgical procedures.

## Discussion

We performed endoscopic PITA in 10 pediatric patients with pediatric OSA who were considered to be at high risk, and none experienced perioperative complications or posterior hemorrhage. 

In pediatric OSA, the airway is obstructed, especially by adenoids and enlarged palatine tonsils, causing not only sleep disturbances but also physical effects such as poor concentration due to daytime sleepiness and learning disabilities. Surgical treatment is indicated based on a comprehensive evaluation of the apnea severity, respiratory status, and physical development. The number of surgeries for obstructive apnea is increasing annually, while the age at indication is decreasing [11].

Tonsillectomy, where the entire capsule of the palatine tonsils is removed, has traditionally been performed, and patients should be aware of pain, decreased intake, and postoperative bleeding due to the surgery. The frequency of postoperative bleeding varies widely, ranging from 1 to 7%, and is independent of the surgeon’s experience [12]. When considering age as a postoperative risk factor, an increased risk of dehydration and postoperative bleeding was reported in children younger than three years [13].

Recently, with the advent of endoscopes and surgical devices, powered intracapsular tonsillectomy, in which the intracapsular tissue is removed using a powered device, has been used in tonsil surgery. Intracapsular tonsillectomy was first reported in 2003 as a less invasive surgical technique, and various reports have been published on its efficacy and safety [14-16]. For example, in 174 pediatric patients with OSA, postoperative bleeding, pain, and food intake significantly improved compared with those after conventional tonsillectomy [9].

However, there are few reports on tonsillectomy in high-risk patients despite the fact that Trisomy 21 [17], cerebral palsy [18], and Prader-Willi syndrome [19] are associated with higher rates of OSA. This could be because tonsillectomy is associated with higher perioperative and postoperative risks even when accounting for the effects of anesthesia and may not improve apneic symptoms. As a high-risk group, the rate of complications is higher in complex pediatric chronic conditions, and the postoperative mortality rate is approximately 30 times higher than that in healthy children, as reported by Edmonson et al., who also examined the postoperative OSA risk of tonsillectomy in patients with Trisomy 21 and found that postoperative OSA persisted [4,20].

In this context, endoscopic PITA was used in 10 cases of OSA in children who were considered high-risk cases; however, none of them showed postoperative hemorrhage. Unlike conventional tonsillectomy, intracapsular tonsillectomy is performed within the capsule, and there is a low risk of injury to the large anatomical blood vessels. The use of an endoscope in conjunction with PITA allows resection to be performed with a clear field of view, enabling the surgeon to observe even slight bleeding during the procedure, which is also thought to allow for accurate hemostasis. Particularly in high-risk cases, the effects of asphyxia and blood loss are significant in cases of postoperative bleeding, and endoscopic PITA is useful for reliable hemostasis. The mean operative time was 61 min. In previous studies, the average time reported was 65.7 min, and there did not appear to be any particular trend toward longer times [9]. The small size of the mouth and face of pediatric patients makes it difficult to secure the visual field with the naked eye and manipulate it using instruments. The use of an endoscope allows resection to be performed with a clear field of view, leading to a safe and reliable operation.

The mean length of hospitalization was 8.8 days. Compared with previous reports, where the average length of stay was 7.27 days, this was approximately one day longer [9]. The criteria for hospitalization varied among facilities, but the decision to discharge was made based on the patient’s overall condition, dietary intake, wound condition, and complaints. In complicated cases, it was difficult to confirm the patient’s complaints and wounds in the mouth after surgery, which may have made the decision to discharge the patient from the hospital difficult; therefore, the period of time may have been slightly longer.

In the high-risk group, there were cases of neurological disease and maxillofacial morphological abnormalities. There is a risk that surgery will not improve apneic symptoms due to other possible causes besides OSA. When deciding on surgery, perioperative risks such as bleeding and pain in addition to postoperative functional risks should be fully considered.

As a limitation of this study, we demonstrated the efficacy of PITA for pediatric complications in 10 cases, but the number of cases is limited, and clinical studies based on more cases are needed. In addition, because this study did not directly compare conventional surgical procedures, it cannot be concluded that PITA is a procedure that must be performed for all pediatric patients with apnea with high-risk complications. In the case of sleep apnea in adults, the posterior airway space, mandibular plane to the hyoid bone, and other anatomical factors have been reported to be related, although they could also be used, and further investigation is needed [21,22].

## Conclusions

This paper describes the use of PITA for treating pediatric patients with OSA, who are considered a high-risk group with complications and difficult perioperative management. To date, there have been no reports of surgery in pediatric patients with OSA with high-risk comorbidities. The data obtained through this study show that an endoscopic PITA approach could reduce the risk of severe bleeding and relieve sleeping conditions in pediatric patients with CCCs.
